# Single Molecule PCR Reveals Similar Patterns of Non-Homologous DSB Repair in Tobacco and *Arabidopsis*

**DOI:** 10.1371/journal.pone.0032255

**Published:** 2012-02-28

**Authors:** Andrew H. Lloyd, Dong Wang, Jeremy N. Timmis

**Affiliations:** School of Molecular and Biomedical Science, The University of Adelaide, South Australia, Australia; Centro de Investigación y de Estudios Avanzados del IPN, Mexico

## Abstract

DNA double strand breaks (DSBs) occur constantly in eukaryotes. These potentially lethal DNA lesions are repaired efficiently by two major DSB repair pathways: homologous recombination and non-homologous end joining (NHEJ). We investigated NHEJ in *Arabidopsis thaliana* and tobacco (*Nicotiana tabacum*) by introducing DNA double-strand breaks through inducible expression of I-SceI, followed by amplification of individual repair junction sequences by single-molecule PCR. Using this process over 300 NHEJ repair junctions were analysed in each species. In contrast to previously published variation in DSB repair between *Arabidopsis* and tobacco, the two species displayed similar DSB repair profiles in our experiments. The majority of repair events resulted in no loss of sequence and small (1–20 bp) deletions occurred at a minority (25–45%) of repair junctions. Approximately ∼1.5% of the observed repair events contained larger deletions (>20 bp) and a similar percentage contained insertions. Strikingly, insertion events in tobacco were associated with large genomic deletions at the site of the DSB that resulted in increased micro-homology at the sequence junctions suggesting the involvement of a non-classical NHEJ repair pathway. The generation of DSBs through inducible expression of I-SceI, in combination with single molecule PCR, provides an effective and efficient method for analysis of individual repair junctions and will prove a useful tool in the analysis of NHEJ.

## Introduction

DNA double strand breaks (DSBs) that occur frequently in eukaryotes are potentially lethal to the cell as they lead to mitotically unstable acentric chromosome fragments and the consequent loss of essential genes [1]. In order to deal with these dangerous cellular lesions several DNA repair pathways exist. When a homologous template is available, DNA repair may occur *via* homologous recombination (HR) [2]. During HR any sequence information lost as a result of DNA damage or degradation at the break site, is recovered by using the homologous chromosome or a sister chromatid as template for repair [3]. DNA DSBs may also be repaired without the use of a homologous template by using non-homologous end joining (NHEJ) [1]. In plants this latter pathway appears responsible for the majority of DSB repair [4]. Classical-NHEJ involves the ku70/ku80 heterodimer which binds to DNA ends [5] and recruits a number of other proteins including the DNA ligase IV/XRCC4 complex which repairs the break [6]. The term alternative-NHEJ (alt-NHEJ) is generally used to describe any NHEJ event which lacks one or more of the core classical NHEJ proteins e.g. ku70, ku80, Lig4, XRCC4 [1]. Alt-NHEJ, sometimes referred to as backup-NHEJ (B-NHEJ) [7] or micro-homology-mediated end joining (MMEJ) [8], is not as well characterised and may well include several distinct repair pathways [1]. It has been suggested that alt-NHEJ is inhibited by classical-NHEJ [9], [10]. Recently, there has been increased research into NHEJ in mammalian systems, as its importance with regard to cancer treatment has become clear. NHEJ promotes cancer cell survival [11] and inhibitors of NHEJ can be used to increase the sensitivity of tumours to DNA damaging drugs [12] or radiation treatment [13].

While NHEJ research is less advanced in plants there is considerable interest in the process, as it is considered the major pathway for transgene insertion by particle bombardment, *Agrobacterium* and zinc-finger nuclease mediated transformation [14], [15], [16] and also for the insertion of cytoplasmic organellar DNA [17], [18]. A better understanding of this pathway may lead to development of more advanced transformation techniques and manipulation of the pathway may enable efficient gene targeting by HR in higher plants [19].

Despite the large body of work investigating NHEJ there are still several shortcomings in the analysis of this form of DSB repair. A number of these arise from the necessity for tissue culture selection to generate clonal cell lines arising from cells that have undergone individual repair events. The requirement for cell culture restricts analysis to tissues or cell lines able to be cultured efficiently, preventing investigation of NHEJ in some tissues of interest. In addition, the selection and maintenance of multiple cell lines is not only labour intensive and time-consuming, but it also hinders analyses needed to uncover subtle variations in NHEJ repair, and to observe rare classes of repair. These problems require the development of a high-throughput pathway for the analysis of NHEJ repair events without the need for rounds of tissue culture, selection and plant regeneration.

Over the past decade single molecule (sm) PCR has become a powerful method for examining DNA sequences at the single cell level. It has been used previously in wide ranging applications [20], [21], [22] and is ideally suited to the analysis of somatic mutations as it allows amplification of a target locus from unique template DNAs [23]. Therefore this technique provides a tool with which to investigate DSB repair, enabling rapid amplification and sequencing of individual repair junctions. We have used smPCR to investigate NHEJ using a genetic system that allows induction of DNA double-strand breaks at a specific nuclear location *in planta*, followed by amplification of individual NHEJ repair junctions. We validated the use of this system in both *Arabidopsis* and tobacco, revealing similar patterns of NHEJ repair in both species and finding insertions at repair sites in *Arabidopsis* in contrast to previous studies [24]. In addition, we demonstrated that sequence insertions at sites of DSB repair in tobacco involve a non-classical NHEJ repair pathway.

In the course of this work we also evaluated the use of the *dao1* dual selectable marker gene [25] in tobacco. While both positive and negative selection worked well in seedling selection experiments, we caution that only positive selection was found to be reliable during tissue culture regeneration.

## Results

### The experimental system

**Figure 1:**
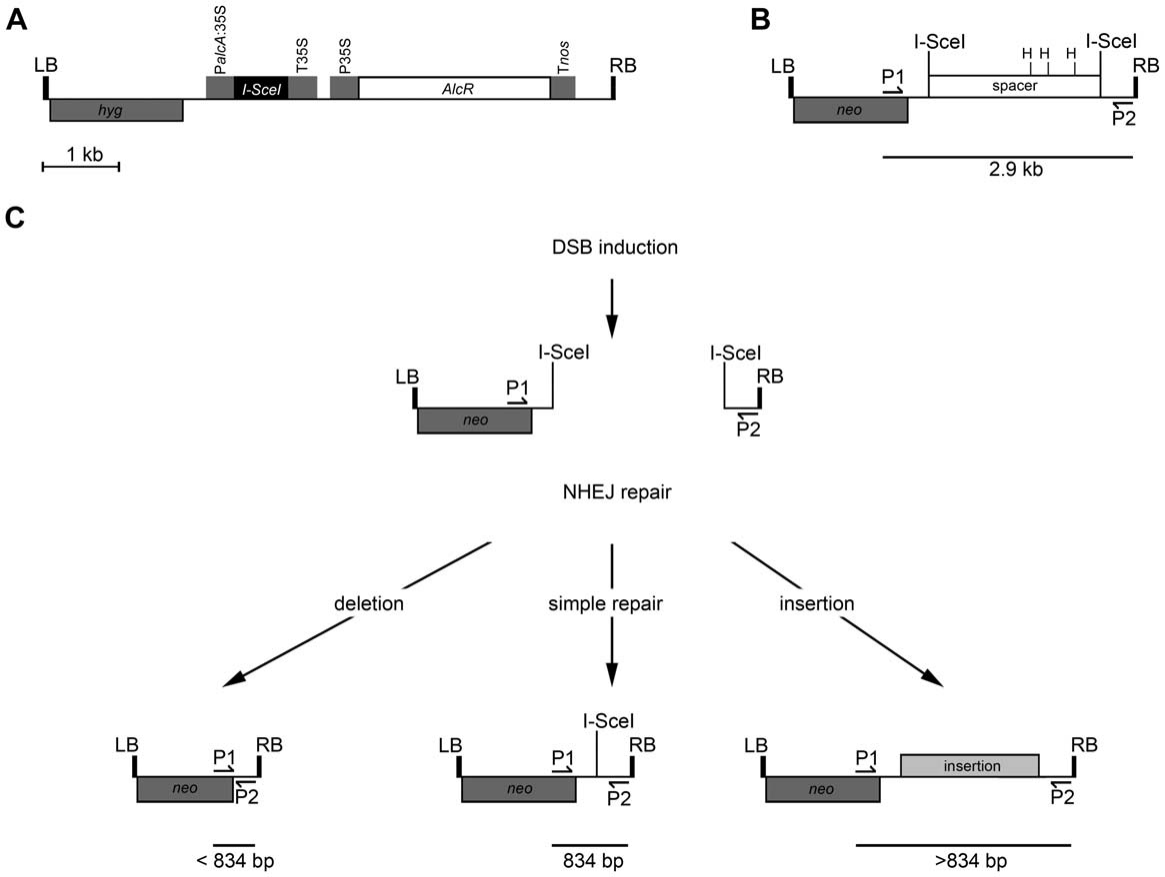
Overview of EtOH induced induction of DSBs. The T-DNA of vector pAlcR:ISceI (**A**) contains the left (LB) and right border (RB) sequences; a hygromycin selectable marker gene (*hyg*); the *AlcR* gene constitutively expressed from the 35S promoter; and the *I-SceI* gene driven by the *alcA*:35S promoter. In the presence of EtOH, AlcR binds to and transcriptionally activates the *alcA*:35S promoter, driving expression of *I-SceI*. The T-DNA of vector pdao1 (**B**) contains left and right border sequences; a kanamycin selectable marker gene (*neo*); and a spacer region flanked by I-SceI target sites. The spacer region also contains three HincII sites (H). (**C**) Upon I-SceI expression the I-SceI sites are cleaved leading to the excision of the spacer region. DSB repair will then result in the joining of the cleaved sequences. This may result in direct joining of the I-SceI restriction sites, deletion of sequence on either side of the DSB or insertion of sequence at the site of DSB repair. These three types of repair can be distinguished by PCR using primers P1 (DSBF1) and P2 (DSBR1) that flank the site of DSB repair. Direct joining will result in an 834 bp product whilst deletion will result in a smaller product and insertion in a larger product. In the absence of DSB induction or when DSBs are repaired by HR the spacer region will not be excised resulting in a PCR product of 2.9 kb (**B**). Amplification of this product will be prevented by digesting template DNA with HincII.

The experimental system consists of a gene encoding the rare cutting endonuclease I-SceI under the control of an ethanol inducible promoter (Figure 1A) [26]. At a second locus two I-SceI restriction sites flank a ∼3 kb ‘spacer region’ (Figure 1B) containing the *dao1* dual selectable marker gene [25] that includes three HincII sites.

To generate DNA DSBs, tissues of interest in a transgenic plant hemizygous for both experimental transgene cassettes (Figure 1) were sprayed with ethanol to induce I-SceI expression which then introduced DSBs at the two restriction sites flanking the spacer region. Following DSB induction the plants were left for several days to allow DSB repair to take place, after which the tissue was harvested and DNA prepared. Individual junctions that had been repaired by NHEJ were then specifically amplified by single molecule PCR using primers flanking the two I-SceI sites. As each product was generated from a single template molecule, every amplicon represents a unique repair junction. Template molecules which contain the spacer region may have been repaired through homologous recombination using the sister chromatid as a template or, alternatively, these molecules may come from cells in which DSBs were not induced. These non-NHEJ template molecules were digested by HincII restriction of the spacer region such that they were not represented in the PCR products (Figure 1B). Repair events that lack the spacer region i.e. those events joining the two I-Sce-I sites to exclude the spacer (Figure 1C), will have arisen through NHEJ, as no chromosomal template molecule exists that is able to mediate such a repair *via* homologous recombination. These NHEJ-derived template molecules are not digested and remain intact, unless a *de novo* insert happens to contain HincII sites.

NHEJ repair junctions that result in no loss of sequence other than the excision of the spacer region will reform one I-SceI site from the two I-SceI half sites generated by the initial DSB induction (Figure 1C). These junctions generate PCR products of 834 bp (Figure 1C). PCR products larger or smaller than this result from NHEJ repair junctions involving insertions or deletions respectively (Figure 1C).

### Generation of experimental lines

To establish the experimental system, two binary *Agrobacterium* transformation constructs, pdao1 and pAlcR:I-SceI, were generated. The pdao1 T-DNA contains *neo* for kanamycin selection and the ‘spacer region’ containing a 35S promoter-driven *dao1* gene flanked by two I-SceI target sites (Figure 1B). The pAlcR:I-SceI T-DNA contains *hyg* for hygromycin selection, *AlcR* driven by a 35S promoter and *I-SceI* driven by the ethanol inducible *aclA*:35S promoter [27] (Figure 1A). The pdao1 and pAlcR:ISceI constructs were individually transformed into *Arabidopsis* and tobacco by *Agrobacterium* transformation to generate D (pdao1) and A (pAlcR:ISceI) lines for both species. For *Arabidopsis* the nuclear location of the pdao1 T-DNA was determined by TAIL-PCR and comparison with the current *Arabidopsis* whole genome assembly (TAIR9).

Antibiotic resistant D and A line shoots (tobacco) or seedlings (*Arabidopsis*) were assayed by PCR to confirm the presence of the pdao1 and pAlcR:ISceI T-DNAs respectively. For PCR positive lines, T_1_ progeny from self fertilised T_0_ plants were grown on the appropriate antibiotic to determine segregation ratios in order to identify lines with single locus T-DNA insertions.

Homozygous, single locus, D line plants were crossed to homozygous, single locus, A line plants to generate doubly hemizygous progeny, providing the starting genotype for DSB induction. The doubly hemizygous lines were designated D4A2 (tobacco) and D19A26 (*Arabidopsis*).

### *dao1* enables dual selection in tobacco seedlings but not in tissue culture

The dual selectable marker gene encoding a D-amino acid oxidase (*dao1*) was included between flanking I-SceI sites to enable selection of seedlings or single cells in tissue culture for both the presence or absence of the spacer region. It was intended that this would be used in a complementary approach to identify NHEJ repair events. Effective use of *dao1* has been demonstrated for both positive and negative selection of *Arabidopsis* seedlings [25] but its use in the selection of tobacco seedlings or in explant shoot regeneration was not previously demonstrated. Experiments showed that *dao1* was effective for use both as a positive and a negative selectable marker gene for selection of germinating seedlings using concentrations of 10 mM D-alanine and 15–30 mM D-valine respectively as the selective agents (Text S1; Figures S1 and S2). In tissue culture, positive selection (D-alanine) but not negative selection (D-valine) was able to clearly distinguish between *dao1* transgenic and wild-type explants. D-valine was therefore unsuitable for negative *dao1* selection in tobacco explant regeneration (Text S1; Figures S3 and S4). As a result *dao1* acts only as the essential spacer DNA in the current experiments.

### Ethanol application leads to I-SceI expression and induction of DSBs

**Figure 2:**
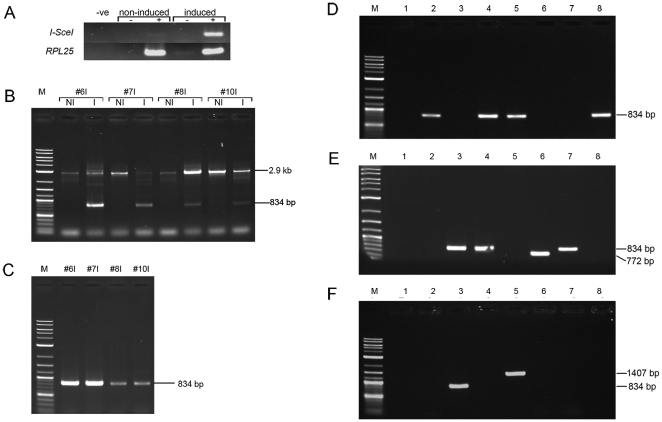
PCR analysis of DSB induction and repair. (**A**) RT-PCR (+) demonstrates increased *I-SceI* mRNA accumulation after induction with 0.7 M ethanol in tobacco leaf tissue. Low levels of I-SceI mRNA accumulate in non-induced leaf tissue. No reverse transcriptase (−) and no template (-ve) controls are shown. Template control RT-PCRs used *RPL25* mRNA primers. (**B**) The DSB region was PCR amplified from 4 tobacco D4A2 plants using primers DSBF1 and DSBR1 which flank the two I-SceI sites. Only the full length 2.9 kb band is amplified from template DNA extracted prior to DSB induction (NI). An additional ∼834 bp band is amplified from template DNA extracted after DSB induction (I). 834 bp is the expected size of the DSB region after excision of the spacer region. (**C**) After HincII digestion of induced template DNA only the 834 bp band is amplified. No amplification is observed from those molecules which have not undergone *dao1* excision. Individual repair junctions were amplified by smPCR (**D**–**F**). The majority of products amplified were ∼834 bp in size. Some repair events resulted in deletions leading to products <834 bp (**E**) while others resulted in insertion leading to products >834 bp (**F**). Examples shown are amplified from D4A2#6I template DNA.

Prior to screening, the efficiency of ethanol induction of *I-SceI* was assessed. Leaf tissue was taken from the tobacco T_0_ A2 plant immediately prior to, and three days after, induction with 0.7 M ethanol. From these tissues RNA was prepared and cDNA synthesised for use in RT-PCR. A very faint gel band was observed for *I-SceI* mRNA prior to induction in leaf tissue (Figure 2A) indicating minimal leaky transcription in the absence of ethanol. After induction a strong band was observed (Figure 2A), indicating a marked increase in transcript accumulation in the presence of ethanol.

Standard PCR was used initially to confirm that I-SceI expression resulted in the induction of DSBs and excision of the spacer region by NHEJ repair. DNA was prepared from tobacco leaf samples taken from experimental lines prior to and four days after ethanol induction. The DSB repair locus was then amplified using primers flanking the two I-SceI sites. A 2.9 kb band was expected from template molecules that had not undergone spacer excision or were repaired by HR (Figure 1B). An 834 bp band was expected from template molecules arising from NHEJ repair of DSBs without any associated insertion or deletion (Figure 1C).

A 2.9 kb band, resulting from amplification of the locus without *dao1* excision, was observed for all DNA templates (Figure 2B). In addition a ∼834 bp product, reflecting excision of the spacer region, was amplified when using template DNA from I-SceI induced tissue (Figure 2B). As both 2.9 kb and ∼834 bp bands were present, it is evident that some template molecules originated from cells where DSBs were repaired by NHEJ and others were from cells repaired by HR (or where DSBs were not induced). Equivalent results indicated that DSB induction was efficient in *Arabidopsis* (data not shown).

### HincII digest allows preferential amplification of junctions repaired by NHEJ

To favour amplification of repaired junctions arising specifically from NHEJ of DSBs, template samples were predigested with HincII which cuts three times within the spacer region but not in the flanking sequences between the I-SceI sites and primer binding sites (Figure 1B). Unavoidably digestion would also prevent amplification of template molecules arising from DSB repair events involving insertion of DNA containing HincII site(s). After HincII digestion only ∼834 bp products were amplified (Figure 2C).

### Single molecule PCR

To amplify PCR products representing individual DSB repair junctions smPCR was used. For smPCR the template DNA was diluted until about two thirds of samples received no template molecules at all. This ensured that ∼80% of the reactions that did generate a PCR product did so from a single template molecule [28]. It was not possible to calculate the average template molecules in a given weight of DNA, as HincII digestion reduced the number of template molecules per unit weight DNA in a manner dependent upon the unknown efficiency of excision of the spacer region. Instead, a series of DNA concentrations were tested to arrive at the desired empirical concentration.

In tobacco, two independent doubly hemizygous plants (D4A2#2I and D4A2#6I) were tested using an optimised DNA concentration of 110–130 pg per reaction. This resulted in a product being amplified in 33–38% of reactions. For both plants this corresponds to one template molecule in ∼275 pg of genomic DNA, or one DSB repair by NHEJ in every 24 genomes. In *Arabidopsis*, one plant (D19A26#1I) was tested and a DNA concentration of 1 pg/reaction was chosen. This resulted in a product being amplified in 27% of reactions, corresponding to one template molecule in ∼3.7 pg, or, one DSB repair by NHEJ in every 9 genomes [29]. These results clearly indicate efficient induction of DSBs and subsequent NHEJ repair.

### Arabidopsis and tobacco have similar patterns of non-homologous DNA repair

389 and 311 unique repair junctions were amplified in tobacco and *Arabidopsis* respectively. The majority of PCR products were ∼834 bp in length (Figure 2D): the size expected with simple joining of the two I-SceI half sites (Figure 1C). For both species ∼1.5% of PCR products were significantly smaller, corresponding to large (>50 bp) deletions (one example is shown in Figure 2E) and ∼1.5% were significantly larger indicating net insertions (one example is shown in Figure 2F). Deletions that resulted in the loss of one or both primer binding sites would not have been observed in this analysis such that a maximum symmetrical deletion size of ∼750 bp could be amplified by PCR using these primers. As a result, 1.3–1.5% is a minimum estimate of the proportion of repair events that involve large deletions. In addition amplification of junction sequences involving insertions would not be possible if the insert was too large for PCR or if the insert contained a HincII restriction site. 1.5% is therefore a conservative estimate of proportion of repair events involving insertion. Occasionally, a 2.9 kb product was amplified from a template molecule containing the spacer region which had not been digested by HincII (data not shown) demonstrating that inserts of at least 2 kb could be amplified effectively using this method.

**Figure 3:**
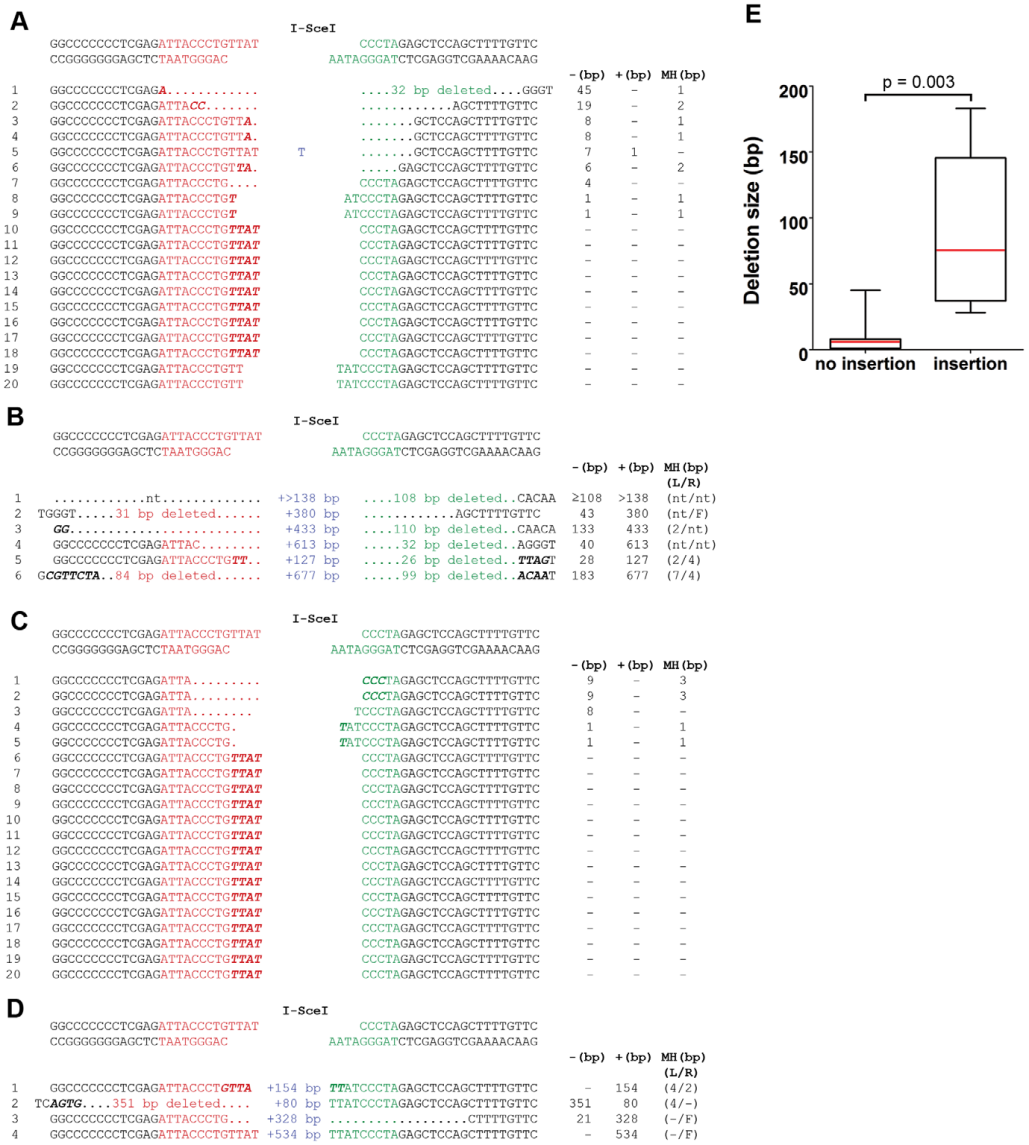
Sequence of double strand break repair events. The sequence surrounding the junction sites is shown for 20 randomly chosen repair products (tobacco, **A**; *Arabidopsis*, **C**), and the repair products that harboured insertions (tobacco, **B**; *Arabidopsis*, **D**). The original sequence generated by I-SceI cleavage is shown at the top of **A–D** (the sequence of both strands is shown). Bases from the I-SceI site upstream of *dao1* are shown in red, bases from the I-SceI site downstream of *dao1* are shown in green. Inserted bases are shown in blue. In some instances microhomology was observed between the terminal bases of the fragments being joined (bold italics). Columns to the right of **A–D** indicate the total length of deletion (**−**), insertion (+) and microhomology (MH, not including I-SceI site overlap), nt signifies not testable, F indicates the insertion of filler DNA. Numbers in brackets indicate length of deletion or microhomology observed at the junction either upstream (L) or downstream (R) of the insertion. In tobacco the median deletion size was considerably larger in DSB repair events involving insertion than in repair events not involving insertion (**E**). The box-and-whisker plot shows the median (red line), the first and third quartiles, and the upper and lower limits of the length of deletions (two-tailed Mann-Whitney U test).

For both plant species, all the insertion events recovered together with 20 randomly chosen smPCR products were sequenced. The smPCR products sequenced that were clearly the result of reactions containing more than one template molecule (∼20%) were discarded. In tobacco and *Arabidopsis* respectively, 45% (9/20) and 75% (15/20) of repair junctions without insertion resulted in no loss of sequence due to simple ligation of the two I-SceI half sites (Figure 3A and 3C) excluding the spacer region. Comparable experiments in mammals showed a similar percentage (40%) of I-SceI site reformation [30]. Two junctions in tobacco were joined by inexact ligation of the two 4 bp I-SceI half site overhangs, resulting in the addition of an extra nucleotide. The remaining junctions, 45% (9/20) for tobacco and 25% (5/20) for *Arabidopsis* had small (1–45 bp) deletions (Figure 3A and 3C). In some instances micro-homology was observed between the terminal bases of the fragments being joined (Figure 3A and 3C) although there was no indication that the amount of micro-homology observed was greater than that expected by chance.

In both tobacco and *Arabidopsis* the average deletion size (∼14 bp and ∼9 bp respectively) was much smaller than the average insertion size (∼95 bp and ∼274 bp respectively). However, as deletions occurred far more frequently than insertions, there was no net loss or gain of sequence at sites of DSB repair in either species.

### Sequences inserted at sites of DSB repair are nuclear in origin

The six insertions in tobacco ranged from 127–677 bp in length (Figure 3B; Table 1) and in all cases insertion was accompanied by deletion of the starting sequence (Figure 3B). Part of insertion NTI1 shared 97% identity with the *Arabidopsis* isoleucine tRNA gene suggesting that it may be SINE-derived sequence [31]. All other inserts showed partial identity to uncharacterised EST clones from tobacco or other Solanaceous species (Table 1), indicating that all insertions were probably of nuclear origin. The four insertions in *Arabidopsis* ranged from 80 to 534 bp in length (Figure 3D; Table 2). Insertion ATI1 originated from an intergenic region on chromosome 1. The DSB locus in line D19 is located on chromosome 5 indicating that insertion ATI1 did not originate from an adjacent or remote syntenic region. Insertion ATI2 was accompanied by a large deletion upstream of the left hand I-SceI site (Figure 3D). The insert was derived from part of this deleted region but was inserted in the opposite polarity. ATI3 also originated from DNA found upstream of the left hand I-SceI recognition sequence (−33 to −352). This sequence was inserted in the same orientation as the original sequence, effectively generating a tandem duplication. Insertion ATI4 was derived from the region excised between the two I-SceI sites. This 498 bp section of the spacer region did not contain any HincII sites, enabling this junction to be amplified by smPCR. This observation implies that similar insertions of segments of the spacer region may have occurred but were missed in this screen through HincII digestion.

**Table 1:** Origin of tobacco insertions.

Insertion	Length	Origin	Accession
NTI1	>138	isoleucine tRNA gene	partial match to AC009755 (97%, e = 2×10^−18^)
NTI2	379	unknown nuclear	partial match to AM843263 (78%, e = 6×10^−57^)
NTI3	430	unknown nuclear	partial match to EB695504 (97%, e = 2×10^−38^)
NTI4	127	unknown nuclear	complete match to FS392274 (99%, e = 2×10^−56^)
NTI5	613	unknown nuclear	partial match to BP133287 (92%, e = 3×10^−18^)
NTI6	677	unknown nuclear	partial match to FN014067 (90%, e = 2×10^−96^) and partial match to AM847760 (80%, e = 6×10^−53^)

**Table 2:** Origin of *Arabidopsis* insertions.

Insertion	Length	Origin
ATI1	154	chromosome 1 (12,474,035…12,474,189)
ATI2	80	DSB left flanking region (−39…−133)^1^
ATI3	328	DSB left flanking region (−352…−33)^1^
ATI4	534	spacer region (+5…+502)^1^

1 co-ordinates given are relative to the left hand I-SceI cleavage site.

In both *Arabidopsis* and tobacco, short stretches of filler DNA (1–36 bp) were inserted at some junctions (Figure S5). Filler DNA was usually derived from a short stretch, or multiple stretches, of nearby sequence that probably primed ligation (Figure S5). Filler DNA has been associated previously with the insertion of T-DNA [32] and organelle DNA [18] suggesting that both integrate during repair of nuclear DSBs.

### Insertion at sites of DSB repair in tobacco is associated with genomic deletion and increased micro-homology at the sequence junctions

In tobacco, the median size of deletion was found to be significantly larger in DSB repair events involving insertion than in those that did not (Figure 3E, p = 0.003, two-tailed Mann-Whitney U test). DSB repair events that resulted in direct ligation of the two I-SceI half sites where excluded from this analysis as the two I-SceI half sites have complementary 4 bp single stranded overlaps that may promote joining without deletion. In addition, only those DSB repair events harbouring insertions >1 bp were included.

Investigation of the presence of micro-homology at repair junctions involving insertion was possible for six junctions. At these, the bases flanking the insert sequence in its original context could be inferred from the EST sequence to which the insert matched. For the other junctions, BLAST searches only identified accessions with limited identity to the insert sequence, preventing unequivocal assessment of micro-homology. This is a limitation of analyses such as this where the sequence from which the insert originates is unknown. Five of the six junctions that could be assessed showed micro-homology (2–7 bp, Figure 3B). The remaining junction showed a 1 bp insertion of filler DNA (Figure 3B, Figure S5). Overall the level of micro-homology observed in insertion repair events was greater than that expected by chance (p = 0.048, n = 6, two-tailed Mann-Whitney U test). The presence of large deletions and micro-homology at repair junctions is indicative of insertion via MMEJ or SDSA.

## Discussion

DSBs have a number of different causes including reactions with oxygen free radicals generated during aerobic respiration, ionizing radiation and faulty action of nuclear enzymes [1]. To deal with these cellular lesions a highly flexible pathway of NHEJ repair has evolved, which enables efficient joining of the many types of damaged DNA ends generated by DSBs. In addition to the mechanistic flexibility of some of the proteins involved [33], NHEJ exhibits multiple levels of redundancy enabling it to function even when some components are lacking [7], [34]. Whether these alternative forms of repair constitute distinct pathways, or are essentially the same flexible pathway with one or more enzymes being substituted, is still unclear [1], [10].

We developed a genetic system that allows induction of DSBs at a known nuclear locus through ethanol inducible expression of the rare cutting endonuclease I-SceI. This approach allowed individual NHEJ repair junctions to be efficiently amplified by smPCR. Using this system we observed a total of ∼700 unique NHEJ junctions in *Arabidopsis* and tobacco, facilitating comparison of NHEJ repair in these two plant species.

In general, *Arabidopsis* and tobacco were found to have very similar patterns of NHEJ repair. In both species, the vast majority of repair events resulted in relatively conservative repair with either no loss of sequence (*At*, 75%; *Nt*, 55%) or small deletions (*At*, 25%; *Nt*, 45%) at the repair junction. In a small percentage of junctions, repair was less conservative and involved large deletions or insertions. Although the average insertion size was greater than the average deletion size, the greater frequency of deletions meant that there was no net loss or gain of sequence at sites of DSB repair. Overall this picture is similar to that observed in mammalian NHEJ [30], highlighting the high degree of conservation in this important pathway.

Our findings provide a similar general picture of NHEJ in the two species which contrasts markedly with previous comparisons that uncovered large differences between specific types of NHEJ repair in *Arabidopsis* and tobacco. Kirik *et al.* [24] investigated NHEJ repair events associated with deletions and observed insertions at a high proportion of repair junctions in tobacco, whereas deletions were larger and insertions were entirely absent in *Arabidopsis*. It is clear from our more extensive results, however, that insertion during NHEJ repair occurs at a similarly low frequency in both tobacco and *Arabidopsis*.

These apparently contradictory findings are best explained by our observation that insertion events in tobacco are associated with high levels of micro-homology and large deletions. The earlier study, by only observing NHEJ events associated with deletion (which was necessary to eliminate the activity of a negative selectable marker gene) may have been strongly biased toward observation of insertion events in tobacco.

The presence of micro-homology and increased deletion size during insertion in tobacco indicates that insertion is not mediated by the classical NHEJ repair pathway. There are (at least) two alternative mechanisms that could explain these observations. One possibility is that these sequences are not inserted *per se* but rather copied into the break site by synthesis dependent strand annealing (SDSA) [35], [36]. In this model, 3′ ends generated by the DSB invade a nearby double stranded DNA molecule and short regions of micro-homology prime synthesis along this template. Template jumping to other nearby sequences may then occur resulting in the synthesis of chimeric insertion sequences. Finally, newly generated complementarity is used to bridge the gap to other side of the DSB. The second possibility is that free-floating DNA fragments close to (or recruited to) the DSB site are inserted, with the insert and DSB ends being joined by small regions of micro homology (MMEJ) [37] or by synthesis dependent MMEJ [38]. Almost all sequence outcomes may be explained equally well by both possible pathways making it essentially impossible to distinguish between the two based on the junction sequence alone. The observation that many insertions are derived from sequences close to the site of the DSB, suggests the SDSA model [39]. It is also clear, however, that many insertions (such as T-DNA insertions and the insertion of organelle DNA) are derived from free floating fragments in the nucleus. In these cases it is likely that the DNA ends are treated similarly to those of a chromosomal DSB and enter the DSB repair pathway leading to insertion.

Increased micro-homology and deletion size are also associated with insertion during NHEJ in mammals [10]. Given the wide conservation of this phenomenon, it is surprising that previous analysis has not found deletions during DSB repair to be associated with insertions in *Arabidopsis* [24]. One possibility is that decreased stability of free DNA ends in *Arabidopsis* [40] may result in larger and more frequent deletions during NHEJ repair. If deletions occur frequently at all NHEJ junctions then they would not be differentially associated with insertion events. We observed no difference, however, between the number of deletions in *Arabidopsis* and tobacco. As we were only able to detect deletion events of up to 750 bp, it is possible that larger deletions, which are known to occur in *Arabidopsis* during NHEJ [24], were missed, concealing a higher frequency of deletion. Indeed, 750 bp is the maximum and deletions between the primers must be symmetrical. As soon as one primer site is deleted, it is no longer possible to amplify the target.

Interestingly, there is recent evidence that chromatin state can affect the pathway of DSB repair, with ku-dependent NHEJ occurring preferentially in euchromatin and ATM mediated DSB repair occurring in heterochromatic regions [41]. ATM is both recruited by [42] and essential for [43] normal DSB repair by the MRN complex which, as well as having a central role in HR [2], is involved in MMEJ repair [44], [45]. Given our finding that DNA insertion during repair of DSBs may be mediated by MMEJ, it is possible that insertion events may occur preferentially in heterochromatic regions. This is a possible explanation for the observation that insertions of mobile elements and organelle DNA tend to occur at heterochromatic pericentromeres [46], [47]. Such a bias would minimise the chance of insertion events disrupting genes while maintaining genome stability and avoiding the loss of potentially useful genetic information. Contradictory to this hypothesis, ATM has been found to suppress MMEJ in mammalian cells [48] but this suppression occurred in plasmid re-circularisation assays and is unlikely to be representative of DSB repair in heterochromatin as efficient nuclear repair is dependent upon distinct histone epigenetic marks [49], [50].

### Conclusion

This study has shown smPCR in this transgenic system to be an efficient method for screening large numbers of DSB repair events and has the potential to be used in wide ranging investigations of DSB repair. Analysis of ∼700 DSB repair events were analysed and, in contrast to previously published evidence suggesting differences in DSB repair between *Arabidopsis* and tobacco, the two species displayed similar DSB repair profiles in our experiments. The majority of repair events were essentially conservative resulting in no, or little, loss of sequence at the junction. A small percentage of repair events resulted in larger deletions or insertion. In tobacco, insertions were associated with larger deletions and micro-homology indicative of insertion via MMEJ or SDSA.

## Materials and Methods

### Plant growth and nucleic acid isolation

*Nicotiana tabacum* and *Arabidopsis thaliana* (Col-0) plants were grown either in soil (in pots) or in tissue culture jars containing 0.5×MS salt medium [51] and 0.8% agar (0.5×MS agar). Soil grown plants were grown in a controlled environment chamber with a 14 hr light/10 hr dark and 25°C day/18°C night growth regime. *In vitro* grown plants were grown in a controlled temperature room with a 16 hr light/8 hr dark cycle at 25°C. For *Arabidopsis*, presumed double hemizygous progeny, resulting from crosses between homozygous A and D line plants were initially grown on 0.5×MS agar medium with 50 mg L^−1^ kanamycin and 15 mg L^−1^ hygromycin to confirm the presence of both T-DNAs, before transferring plants to soil. DNA was extracted using a DNeasy Plant Mini Kit (Qiagen, Hilden, Germany) according to manufacturer's instructions, or by phenol/chloroform extraction [52]. RNA was extracted using an RNeasy Plant Mini Kit (Qiagen) according to manufacturer's instructions

### Plasmid construction and plant transformation

#### pAlcR:ISceI

The *AlcR* expression cassette containing the 35S promoter, *AlcR* ORF, and *nos* terminator was isolated as a NcoI/HindIII fragment from pbinSRN [26]. This cassette was blunt ended using the Klenow fragment of DNA pol I and cloned into SmaI cut pGreen0179 to generate pG.AlcR. The *I-SceI* coding region was excised from pCISceI [53] and inserted between the alcA:35S promoter and *nos* terminator in Alc-pUC (kindly provided by Dr V. Radchuk), using BamHI. Primers AlcF_NcoI (TTCCATGGGATAGTTCCGACCTAGGATGG) and AlcR_NcoI (TTCCATGGGGCGATTAAGTTGGGTAACG) were then used to amplify the *I-SceI* expression cassette and the product was ligated into pG.AlcR using NcoI to generate pAlcR:ISceI.

#### pdao1

The 35S terminator from pPRVIIIA::neoSTLS2 [54] and 35S promoter from p35S (kindly provided by Dr S. Delaney) were cloned into pGreen0029 [55] using HindIII/BamHI and NotI/XbaI respectively. The *dao1* coding sequence was amplified from pVC_RLM_1qcz (kindly provided by Dr. A. Renz, BASF Plant Science) using primers dao1F (GAGAAAGGAAGGGAAGAAAGC) and dao1R_XbaI (ACTCTAGACCTACAACTTCGACTCCCG), the PCR product was then digested with XbaI and cloned into the pGreen0029 vector containing the 35S promoter and terminator, thus generating pG.dao1. A multiple cloning site containing two I-SceI restriction sites flanking HindIII and NotI sites was generated by annealing two complementary oligonucleotides I-SceIMCS1 (CTAGGGATAACAGGGTAATAAGCTTGCGGCCGCTAGGGATAACAGGGTAATC) and I-SceIMCS2 (TCGAGATTACCCTGTTATCCCTAGCGGCCGCAAGCTTATTACCCTGTTATCCCTAGAGCT). This double stranded MCS had 4 bp overhangs at each end allowing ligation into SacI and XhoI cut pGreen0029, generating pG.MCS. The *dao1* expression cassette was excised from pG.dao1 with HindIII and NotI and cloned into HindIII/NotI digested pG.MCS to generate pdao1.

Transformation was performed using the pGreen system of binary transformation vectors [55]. Transgenic tobacco lines were generated using a standard leaf disc method [56]. Transgenic Arabidopsis lines were generated using the simplified floral-dip method [57] with a rapid selection protocol [58]. Putative D line and A line transformants were confirmed by PCR using primer pairs dao1F2/dao1R2 (GGCAAACCGTCCTCGTCAAG/TGACCTCCTTCTCCTTCGCC) and AlcRF1/AlcRR1 (CGTCGTTCTTATTCACTCGTTTGC/TTGGAGGATGGGAAATGCGTTAG) respectively.

### Evaluation of *dao1* selection

To evaluate the use of *dao1* as a selectable marker gene, both wild type and *dao1* transgenic seedlings were grown on 0.5×MS agar medium containing various concentrations of D-alanine (positive selection) or D-valine (negative selection). In addition, leaf explants from both wild type and *dao1* transgenic plants were grown on regeneration medium containing various concentrations of D-alanine and D-valine. For full methods see Text S1.

### Experimental induction of DSBs

For RT–PCR DNA was removed from RNA samples using a TURBO DNA-free kit (Ambion, Austin, TX). Reverse transcription was performed using an Advantage RT-for-PCR kit (Clontech, Mountain View, CA) with an oligo(dT) primer in accordance with the manufacturer's instructions. Samples were also prepared without RT. For amplification of *I-SceI*, primers I-SceIF1 (ACAAACTGGCTAACCTGTTCATCGT), and I-SceIR1 (TTCGGAGGAGATAGTGTTCGGCA) were used. RPL25 mRNA was amplified using primers L25F (AAAATCTGACCCCAAGGCAC) and L25R (GCTTTCTTCGTCCCATCAGG). For tobacco *I-SceI* expression was induced in the leaves of one month old D4A2 plants grown in tissue culture jars. Leaves were sprayed with 1–2 mL of 0.7 M ethanol and the jar lids replaced. For *Arabidopsis* one month old plants selected on petri dishes and then grown in soil were sprayed with 1–2 mL of 0.7 M ethanol and covered with a plastic bag to maintain the presence of ethanol vapour. The plants were then left for 4 days to allow time for I-SceI expression, the generation of DSBs and their subsequent repair. After 4 days leaf tissue was sampled.

### PCR

TAIL-PCR was undertaken as described [59] using degenerate primer AD2 [59] and pdao1 T-DNA specific primers dao1T1 (TCTTCCGCTTCCTCGCTCACTGACTCG), dao1T2 (CTCACTCAAAGGCGGTAATACGGTTATCCA) and dao1T3 (CCACAGAATCAGGGGATAACGCAGGAAAG). Standard PCR was performed using taq polymerase (ROCHE, Basel, Switzerland), using suggested PCR conditions. DSB PCR products were amplified with LongAmp taq DNA polymerase (New England Biolabs, Ipswich, MA) using suggested PCR conditions, primers DSBF1 (GATAGTGACCTTAGGCGACTTTTGAACG) and DSBR1 (TCCCCTGATTCTGTGGATAACCGT), an annealing temperature of 59°C and 40 ng template DNA. Non-induced, induced/undigested and induced/digested DNA was used as template. For digested template, 2 µg genomic DNA was digested overnight at 37°C using 20 u HincII (New England Biolabs) in a 20 µL reaction and purified using a PCR purification kit (QIAGEN) according to manufacturer's instructions.

Single molecule PCR was performed using LongAmp taq DNA polymerase and HincII digested DNA as template. Reactions were 2 µL in volume and contained 0.3 mM dNTPs, 0.4 µM primers (DSBF1 DSBR1), 0.2 u LongAmp taq DNA polymerase, 1× LongAmp buffer and 110–130 pg template DNA for tobacco or 1 pg template DNA for *Arabidopsis*. Reactions were overlayed with mineral oil to prevent evaporation. Cycle conditions were as follows: Initial denaturation 95°C 30′ then 45 cycles of 95°C 20′; 59°C 20′; and 65°C 4″ followed by a final extension at 65°C for 10″. After PCR, 18 µL of H_2_O was added to each reaction to give a total volume of 20 µL. 5 µL was analysed by standard agarose gel electrophoresis and the remainder used in subsequent sequencing.

### Statistical and sequence analysis

Statistical analysis of deletion size and use of micro-homology (two-tailed Mann-Whitney U test) was performed using Prism 5 (GraphPad Software). Junction sequences were analysed using Geneious (verion 5.3, Drummond AJ et al. 2010 [http://www.geneious.com/]). BLAST analysis was performed on several databases, including NCBI's non-redundant nucleotide collection (nr/nt) and non-human non- mouse ESTs (est_others).

## Supporting Information

Figure S1 **D-alanine and D-valine are suitable for positive and negative selection of dao1 respectively in tobacco.** Seedlings of transgenic lines containing *dao1* and wild-type (wt) seedlings were grown on various concentrations of D-alanine (**A**) and D-valine (**B**) or media containing neither amino acid (**A–B**). D-alanine was most effective at a concentration of 10 mM leading to a strong reduction in the growth of wt seedlings while not affecting the growth of transgenic seedlings (**A**). D-valine was most effective at a concentration of 30 mM leading to a marked reduction in the growth of transgenic seedlings while not affecting the growth of wt seedlings. 50 mM D-valine was toxic to both transgenic and wt seedlings and wt seedlings grown at this concentration were unable to be distinguished from transgenic seedlings. Error bars for both A and B show SD.
(TIF)

Figure S2 ***dao1*** **transgenic and wild-type seedlings were easily distinguishable by sight when grown on both 10 mM D-alanine and 30 mM D-valine.** *dao1* transgenic seedlings grown on 10 mM D-alanine showed strong growth (**A,C**), wild-type (wt) seedlings grown on the same medium bleached soon after germination (**B,D**). *dao1* transgenic seedlings grown on 30 mM D-valine had reduced growth (**E,G**) although seedlings did not bleach, cotyledons failed to fully expand and there was no growth of the first true leaf, wt seedlings grown on the same medium showed strong growth (**F,H**). Scale bars for A, B, E and F = 5 mm, scale bars for C, D, G and H = 2 mm.
(TIF)

Figure S3 **10 mM D-alanine is suitable for positive selection of tobacco leaf tissue explants but 30 mM D-valine is not suitable for negative selection.** Leaf explants taken from wild-type plants (wt) were killed when grown on regeneration medium containing 10 mM D-alanine (**A**). Resistant shoots were generated from *dao1* positive leaf explants grown on same media (**B**). Leaf explants from both wt and *dao1* positive plants were killed when grown on regeneration medium containing 10 mM D-valine (**E–F**). Scale bar = 10 mm.
(TIF)

Figure S4 **D-valine is not suitable for negative selection of tobacco leaf tissue explants.** At concentrations of both 15 mM and 5 mM D-valine both *dao1* positive and wild-type (wt) explants failed to generate resistant shoots (**A–D**). At a concentration of 2 mM D-valine both *dao1* positive and wt explants (white boxed area) generated shoots (**E**). Scale bar = 10 mm.
(TIF)

Figure S5 **Filler DNA at repair junctions was derived from short stretches of flanking sequence.** Filler DNA (pink) was observed at three sites of DSB repair, each involving an insertion (NTI1, ATI3 and ATI4). The filler DNA found between the insert sequence (blue) and the original DSB locus sequence (black) was derived from short stretches of DNA flanking the junction (underlined, bold). The homology at the filler DNA donor sites (bold) often extends into sequence flanking the filler DNA suggesting that several base pairs of micro-homology were used to prime the synthesis of the filler DNA promoting joining of the loose DNA ends. Numbers in brackets indicate bases missing from the diagram.
(TIF)

Text S1 **Full methods and supporting material.**
(DOC)
