# Supplementary figures and images for: Single Molecule PCR Reveals Similar Patterns of Non-Homologous DSB Repair in Tobacco and Arabidopsis
Source: PLoS One. 2012 Feb 28;7(2):e32255. doi: 10.1371/journal.pone.0032255 (PMC3289645; doi:10.1371/journal.pone.0032255)

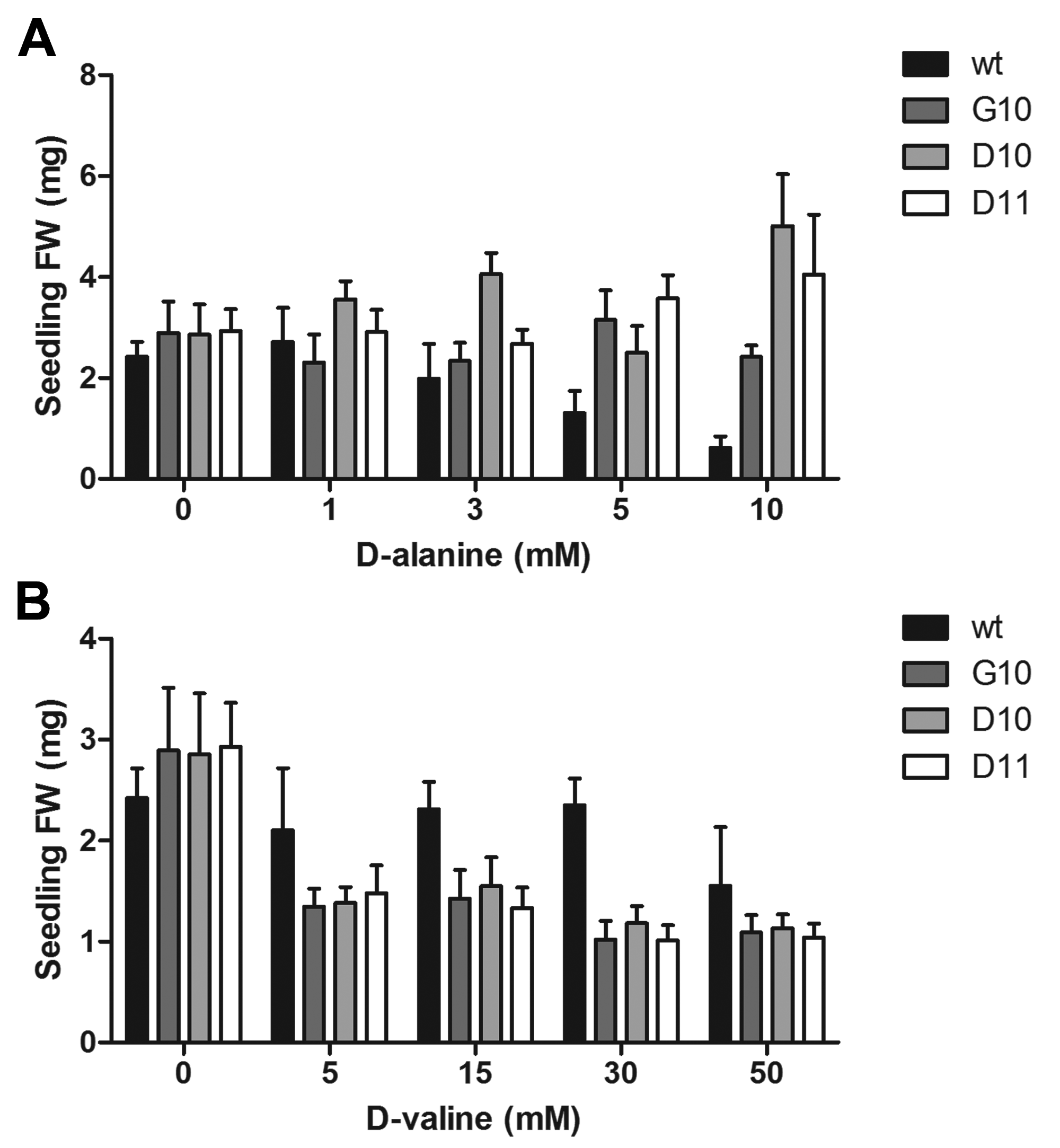

Supplement: Figure S1 — D-alanine and D-valine are suitable for positive and negative selection of dao1 respectively in tobacco. Seedlings of transgenic lines containing dao1 and wild-type (wt) seedlings were grown on various concentrations of D-alanine (A) and D-valine (B) or media containing neither amino acid (A–B). D-alanine was most effective at a concentration of 10 mM leading to a strong reduction in the growth of wt seedlings while not affecting the growth of transgenic seedlings (A). D-valine was most effective at a concentration of 30 mM leading to a marked reduction in the growth of transgenic seedlings while not affecting the growth of wt seedlings. 50 mM D-valine was toxic to both transgenic and wt seedlings and wt seedlings grown at this concentration were unable to be distinguished from transgenic seedlings. Error bars for both A and B show SD. (TIF) [file pone.0032255.s001.tif]

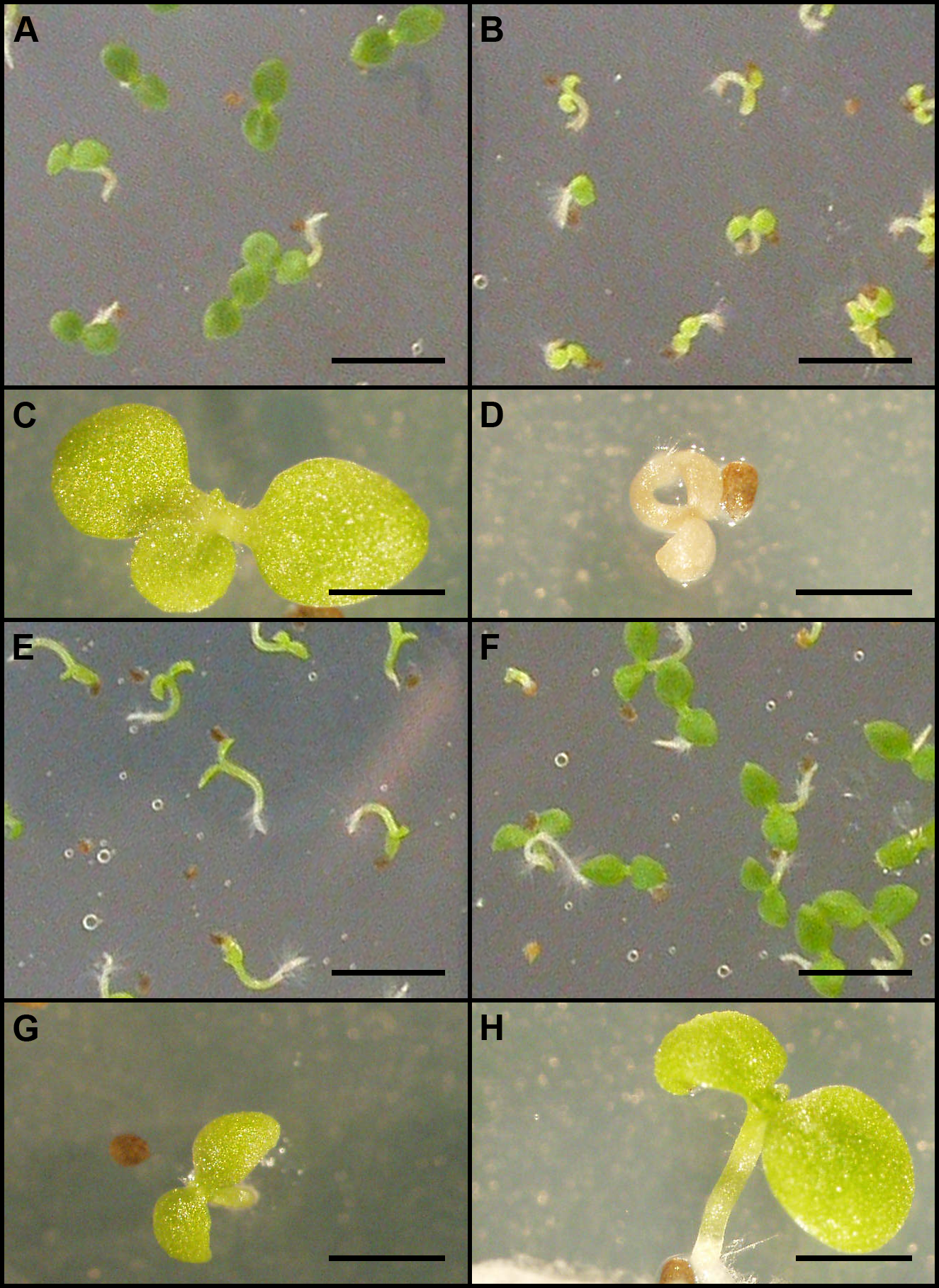

Supplement: Figure S2 — dao1 transgenic and wild-type seedlings were easily distinguishable by sight when grown on both 10 mM D-alanine and 30 mM D-valine. dao1 transgenic seedlings grown on 10 mM D-alanine showed strong growth (A,C), wild-type (wt) seedlings grown on the same medium bleached soon after germination (B,D). dao1 transgenic seedlings grown on 30 mM D-valine had reduced growth (E,G) although seedlings did not bleach, cotyledons failed to fully expand and there was no growth of the first true leaf, wt seedlings grown on the same medium showed strong growth (F,H). Scale bars for A, B, E and F = 5 mm, scale bars for C, D, G and H = 2 mm. (TIF) [file pone.0032255.s002.tif]

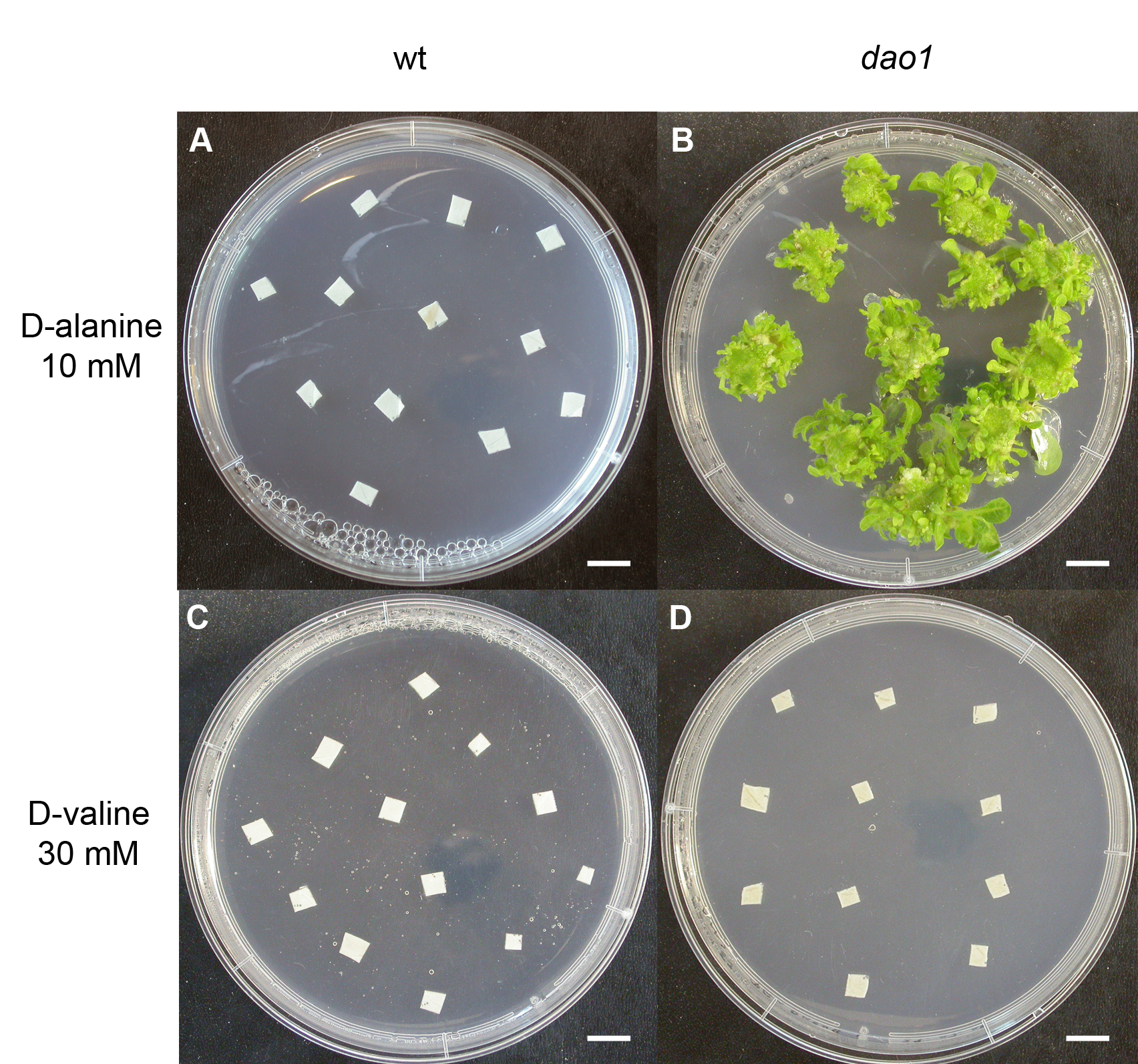

Supplement: Figure S3 — 10 mM D-alanine is suitable for positive selection of tobacco leaf tissue explants but 30 mM D-valine is not suitable for negative selection. Leaf explants taken from wild-type plants (wt) were killed when grown on regeneration medium containing 10 mM D-alanine (A). Resistant shoots were generated from dao1 positive leaf explants grown on same media (B). Leaf explants from both wt and dao1 positive plants were killed when grown on regeneration medium containing 10 mM D-valine (E–F). Scale bar = 10 mm. (TIF) [file pone.0032255.s003.tif]

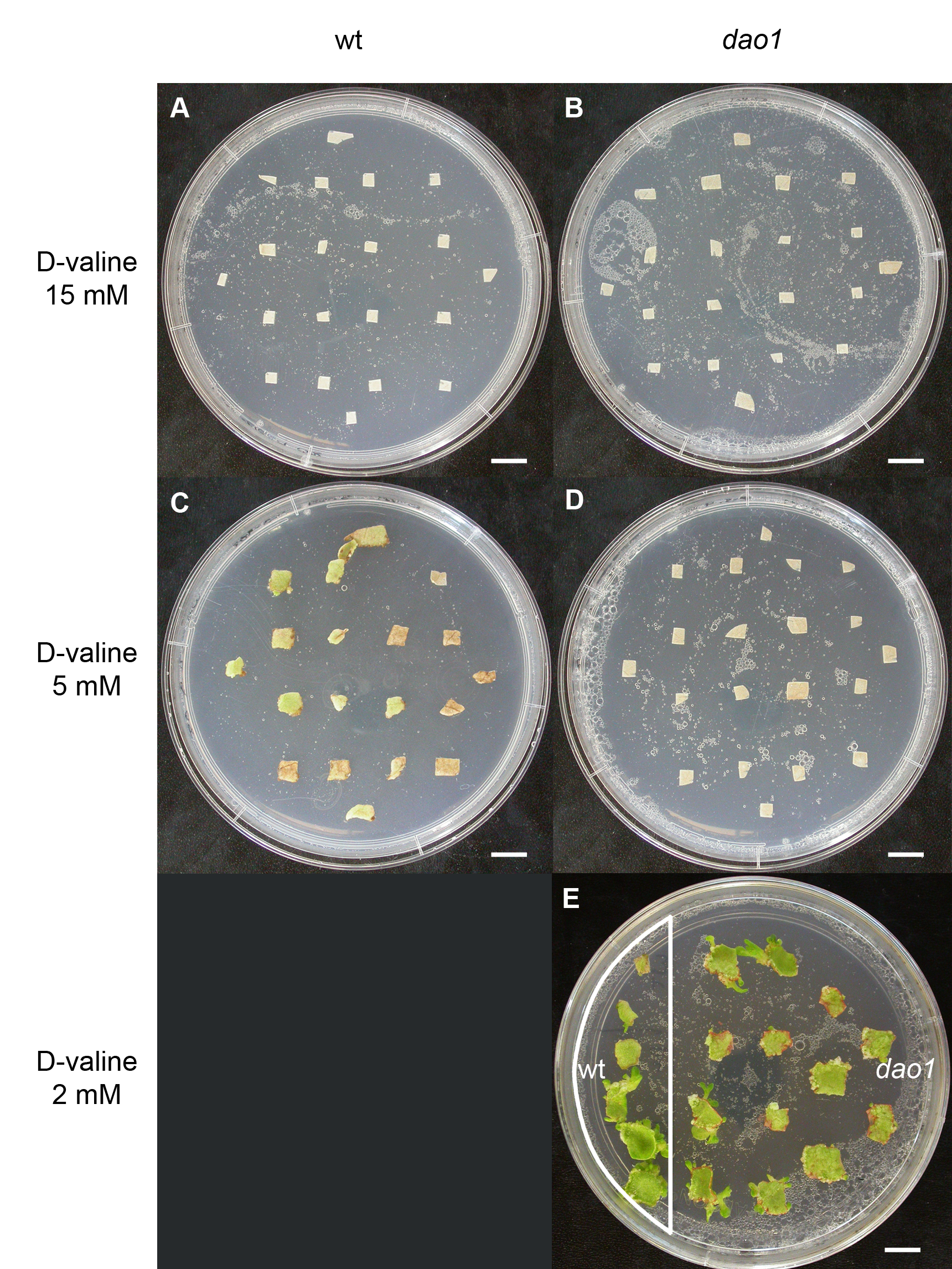

Supplement: Figure S4 — D-valine is not suitable for negative selection of tobacco leaf tissue explants. At concentrations of both 15 mM and 5 mM D-valine both dao1 positive and wild-type (wt) explants failed to generate resistant shoots (A–D). At a concentration of 2 mM D-valine both dao1 positive and wt explants (white boxed area) generated shoots (E). Scale bar = 10 mm. (TIF) [file pone.0032255.s004.tif]

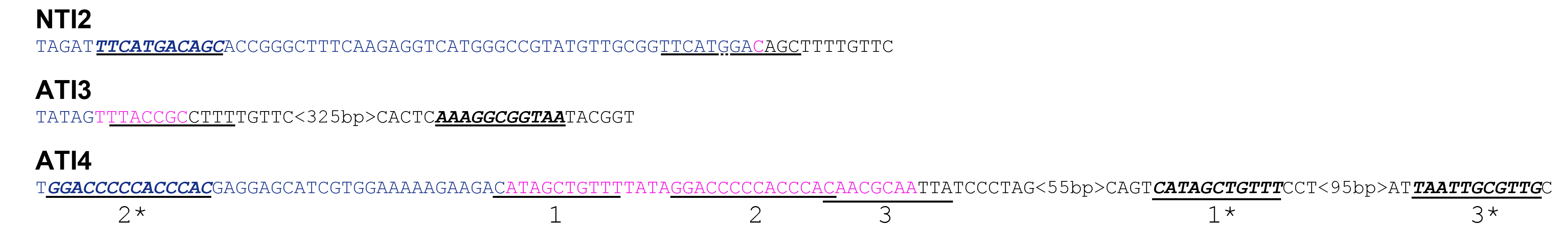

Supplement: Figure S5 — Filler DNA at repair junctions was derived from short stretches of flanking sequence. Filler DNA (pink) was observed at three sites of DSB repair, each involving an insertion (NTI1, ATI3 and ATI4). The filler DNA found between the insert sequence (blue) and the original DSB locus sequence (black) was derived from short stretches of DNA flanking the junction (underlined, bold). The homology at the filler DNA donor sites (bold) often extends into sequence flanking the filler DNA suggesting that several base pairs of micro-homology were used to prime the synthesis of the filler DNA promoting joining of the loose DNA ends. Numbers in brackets indicate bases missing from the diagram. (TIF) [file pone.0032255.s005.tif]
