# Supplementary material for: Single Molecule PCR Reveals Similar Patterns of Non-Homologous DSB Repair in Tobacco and Arabidopsis
Source: PLoS One. 2012 Feb 28;7(2):e32255. doi: 10.1371/journal.pone.0032255 (PMC3289645; doi:10.1371/journal.pone.0032255)
Supplement: Text S1 — Full methods and supporting material. (DOC) [file pone.0032255.s006.doc]

**Single molecule PCR reveals similar patterns of non-homologous DSB repair in Tobacco and *Arabidopsis.***

Andrew H. Lloyd, Dong Wang and Jeremy N. Timmis

**Supporting Material Text**

*Results 2*

*Discussion 3*

*Materials and Methods 4*

*References 5*

**Results**

**Additional pdao1 lines**

In addition to the *dao1* lines described in the main text, additional single locus tobacco lines D13 and D14 were also generated.

**Transformation with vector pGU.D.US**

Transformed lines (G lines) were generated *via Agrobacterium* transformation using the transformation vector pGU.D.US. The pGU.D.US vector contains two overlapping sections of the GUS reporter gene (GU and US), between which is located the *dao1* gene flanked by I-SceI sites. G lines were used in experiments to ascertain the effectiveness of *dao1* selection in tobacco but were not used in any of the downstream double strand break experiments. For the purposes of *dao1* evaluation D and G lines can be considered equivalent, i.e. both contain the *dao1* gene. The pGU.D.US transformation vector was transformed into *Nicotiana tabacum* cv. Wisconsin 38 *via Agrobacterium* transformation to generate G lines. Putative transformants were confirmed by PCR.

***dao1* seedling selection**

T1 seedlings of two single locus lines G10 and D11 and one multiple locus line D10 as well as wild-type seedlings were grown on a range of D-alanine concentrations (1 mM, 3 mM, 5 mM and 10 mM), a range of D-valine concentrations (5 mM, 15 mM, 30mM and 50 mM) and on media containing neither D-amino acid. After two weeks seedlings were weighed and assessed for growth. All transgenic lines showed strong growth on all concentrations of D-alanine whilst wild-type showed significantly stunted growth at 5 mM D-alanine and did not grow past germination on 10 mM D-alanine (Fig. S2). Growth at 10 mM D-alanine showed the greatest distinction between transgenic and wild-type seedlings. At this concentration wild-type and transgenic seedlings were easily distinguishable by sight (Fig. S2). Transgenic lines had significantly reduced growth at all concentrations of D-valine tested and were unambiguously distinguishable visually from wild-type at concentrations of 15 mM and 30 mM (Fig. S2). At the highest concentration (50 mM), D-valine became toxic to wild-type seedlings and they were no-longer easily distinguishable from seedlings containing *dao1* (Fig. S1). 15-30 mM D-valine was therefore determined to be the optimum concentration range for *dao1* negative selection in tobacco seedlings. At this concentration wild-type and transgenic seedlings were easily distinguishable by sight (Fig. S2). These findings indicate that *dao1* is suitable for use as both a positive and negative selectable marker gene in tobacco seedlings.

***dao1* explant selection**

The suitability of *dao1* as a marker gene in tissue culture regeneration experiments was also assessed. D line plants and wild-type plants were grown in tissue culture jars. After 4 weeks, leaf explants taken from both D line and wild-type plants were transferred to regeneration medium containing either 10 mM D-alanine or 30 mM D-valine - these being the concentrations identified as optimum for seedling selection. As expected, in the presence of D-alanine (positive selection) shoots were generated from leaf explants containing *dao1* whilst all wild-type explants died (Fig. S3). However, in the presence of 30 mM D-valine (negative selection) both wild-type and transgenic explants failed to grow (Fig. S3). Lower concentrations of D-valine were then tested. At concentrations of 15 mM D-valine and 5 mM D-valine both transgenic and wild-type explants failed to grow (Fig. S4), at 2 mM D-valine shoots were generated from both transgenic and wild-type explants (Fig. S4). Although it is possible that an intermediate D-valine concentration (2 mM < X < 5 mM) might allow wild-type growth but prevent transgenic growth, this would most likely lead to a high rate of false positives and false negatives due to the minimal window of suitable D-valine concentration. D-valine was therefore found to be unsuitable for negative *dao1* selection in explant regeneration.

**Discussion**

Growth of plants on media containing D-alanine and D-serine will select for the presence of *dao1*, whilst growth on media containing D-valine and D-isoleucine will select for the absence of *dao1* . whilst this selectable marker gene has been shown to work in Arabidopsis and Maize it was not known if this marker gene would function in tobacco. Analysis showed that *dao1* was effective for use both as a positive and a negative selectable marker gene for *in vitro* selection of germinating seedlings using concentrations of 10 mM D-alanine and 15-30 mM D-valine respectively as the selective agents. In tissue culture, positive selection but not negative selection was able to clearly distinguish *dao1* transgenic and wild-type explants. D-valine, used in negative selection, is converted to the toxic 3-methyl-2-oxo butanoic acid by *dao1,* however, D-valine is itself toxic when used at high concentrations. There is therefore a “concentration window” at which D-valine concentration is sufficiently low to allow growth of cells lacking *dao1* but sufficiently high to allow the production of phytotoxic levels of 3-methyl-2-oxo50 butanoic acid in cells containing *dao1*. In seedlings this “concentration window” was determined to be 15-30 mM D-valine, in explant regeneration there does not appear to be a suitable concentration of D-valine that will result in growth of wild-type cells and death of those containing the *dao1* gene. Recently, D-valine was also shown to be ineffective in tissue culture selection of apple calli, being both toxic to wild-type as well as *dao1* transgenic plants . This same study found that wild-type and *dao1* transgenic calli could be distinguished if D-isoleucine was used as the negative selective agent. However, the use of D-isoleucine in regenerating rare cells lacking *dao1* amongst a majority of cells containing *dao1* was not investigated. Based on the findings of Hattasch *et al.* (2009) negative selection may be possible in tobacco using D-isoleucine, however, the cost of D-isoleucine (over 100 times the price of D-valine) may make large scale screens prohibitive.

**Materials and Methods**

**Plasmid construction**

*pGU.D.US*

The GU.C.US region from pGU.C.USB , containing two overlapping regions of the *GUS* gene flanked by a 35S promoter and terminator and separated by a *codA* gene flanked by I-SceI target sites, was excised and cloned into pGreen0029 using BamHI and HindIII to create pGU.C.US. The *dao1* expression cassette was then excised from pdao1 and cloned into pGU.C.US using I-SceI to create pGU.D.US.

***dao1* seedling selection**

Positive and negative *dao1* selection was evaluated by growing T_1_ seedlings from two single locus lines (G10 and D11), one multiple locus line (D10) and wild-type on 0.5 × MS agar medium containing a range of D-alanine concentrations (1 mM, 3 mM, 5 mM and 10 mM), a range of D-valine concentrations (5 mM, 15 mM, 30mM and 50 mM) and on media containing neither D-amino acid. Two weeks after germination the average seedling weight (average fresh weight of 10 seedlings) was determined for each line at each concentration of D-amino acid. At this stage the ability to visually distinguish transgenic and non-transgenic seedlings was also assessed.

***dao1* tissue culture selection**

T_1_ *dao1* line seedlings were grown on media containing kanamycin to select positive segregants and these were transferred to 0.5 × MS agar medium in tissue culture jars for further growth. Wild-type seedlings were sown directly onto 0.5 × MS agar medium in tissue culture jars. After four weeks growth in jars, leaf explants from both wild-type and *dao1*-transgenic plants were placed adaxial side down on regeneration MS104 medium containing D-alanine (10 mM) or various concentrations of D-valine (30 mM, 15 mM, 5 mM and 2 mM).

**References**

**Erikson O, Hertzberg M, Nasholm T** (2004) A conditional marker gene allowing both positive and negative selection in plants. Nature Biotechnology **22:** 455-458

**Hattasch C, Flachowsky H, Hanke MV** (2009) Evaluation of an alternative D-amino acid/DAAO selection system for transformation in apple (Malus X domestica Borkh.). Journal of Horticultural Science & Biotechnology**:** 188-194

**Hellens RP, Edwards EA, Leyland NR, Bean S, Mullineaux PM** (2000) pGreen: a versatile and flexible binary Ti vector for *Agrobacterium*-mediated plant transformation. Plant Mol. Biol. **42:** 819-832

**Lai FM, Mei KF, Mankin L, Jones T** (2007) Application of two new selectable marker genes, *dsdA* and *dao1* in maize transformation. *In* Z Xu, J Li, Y Xue, W Yang, eds, Biotechnology and Sustainable Agriculture 2006 and Beyond: Proceedings of the 11th IAPTC&B Congress. Springer, Dordrecht, pp 141-142

**Mathis NL, Hinchee AW** (1994) *Agrobacterium* inoculation techniques for plant tissues. *In* SB Gelvin, RA Schilperoort, eds, Plant Molecular Biology Manual, Ed 2nd. Kluwer Academic Publishers, Dordrecht, The Netherlands, pp 1-9

**Siebert R, Puchta H** (2002) Efficient repair of genomic double-strand breaks by homologous recombination between directly repeated sequences in the plant genome. Plant Cell **14:** 1121-1131
